# A Low-Cost Digital Colorimetry Setup to Investigate the Relationship between Water Color and Its Chemical Composition

**DOI:** 10.3390/s21206699

**Published:** 2021-10-09

**Authors:** Ruosha Zeng, Chris M. Mannaerts, Zhehai Shang

**Affiliations:** 1 Department of Water Resources, Faculty of Geo-Information Science and Earth Observation, University of Twente, 7514 AE Enschede, The Netherlands; c.m.m.mannaerts@utwente.nl; 2 Institut de la Mer de Villefranche, 181 Chemin du Lazaret, 06230 Villefranche-sur-Mer, France; zhehai.shang@imev-mer.fr

**Keywords:** digital colorimetry setup, water quality, optically active constituents (OACs), color index, citizen science

## Abstract

Developments in digital image acquisition technologies and citizen science lead to more water color observations and broader public participation in environmental monitoring. However, the implications of the use of these simple water color indices for water quality assessment have not yet been fully evaluated. In this paper, we build a low-cost digital camera colorimetry setup to investigate quantitative relationships between water color indices and concentrations of optically active constituents (OACs). As proxies for colored dissolved organic matter (CDOM) and phytoplankton, humic acid and algae pigments were used to investigate the relationship between water chromaticity and concentration. We found that the concentration fits an ascending relationship with xy chromaticity values and a descending relationship with hue angle. Our investigations permitted us to increase the information content of simple water color observations, by relating them to chemical constituent concentrations in observed waters.

## 1. Introduction

Water quality is related to the ecological environment, water cycle dynamics as well as human impacts on water resources. The use of water color as a surrogate or proxy for water quality is rapidly increasing due to the rapid expansion of smartphone applications as citizen tools for observing and monitoring aquatic environments [1,2,3].

Water color is an important and distinct indicator of water quality. The different colors of natural water bodies have been described for centuries, with records tracing back to writings of the 16th century and paintings of the 18th century [4]. Since the 19th century, a color comparator scale called Forel-Ule was adopted by oceanographers to record water color data [5]. It was later converted to a modern tool and connected with chromaticity values (x, y) and dominant wavelengths in 2014 by Novoa et al. [6]. The color of natural waters depends on the optically active components (OACs) in the water, which interact with light photons. The three main OACs in natural waters are phytoplankton pigments, suspended particulate matter (SPM) and colored dissolved organic matter (CDOM). Enhanced phytoplankton concentrations usually result in a green coloration of the water, except for certain species that cause a red or brown color. SPM produces a brownish or reddish color, and CDOM leads to a yellow to brownish color.

At the satellite level, the color of natural waters is typically derived from remote sensing reflectance ($R_{rs}$) of satellites bands and then used to estimate the water quality variables. Different color indices are used for water color classification. For instance, the hue angle derived from low to medium spatial resolution satellite sensors helps in the water quality analysis [7]. The Forel-Ule index, derived from MODIS data, has been used to assess the trophic state of inland waters on a global scale [8]. More attempts to connect color indices with remote sensing reflectance have been implemented. Pitarch et al. [9] has presented 15 years of the evaluation of hue angle and FUI through $R_{rs}$ of oceanic waters on a global scale. Gao et al. [10] developed a method to estimate water leaving reflectance via digital imagery.

On the citizen science side, color indices, including Hue angle, Forel-Ule scale (FU-scale), dominant wavelength and saturation, have been implemented recently in projects that assessed water quality based on digital images collected with smartphone applications. Examples of these smartphone applications are Eyeonwater developed within the European FP7 Citclops project [1] and HydroColor developed by the University of Maine [11]. Both of these applications analyze the photographed natural water and output color indices and water quality indicators. However, both applications have low accuracy for the estimation of Chlorophyll-a and CDOM [12,13].

Color-based measurements, such as colorimetric test strips, have been used in analytical chemistry for decades. The information received by our naked eye usually is hard to quantify without a unit standard. However, with digital-imaging devices such as webcams or built-in smartphone cameras, it is possible to make the color-based measurement more accurate and to provide more details on each color bands (R/G/B) compared to the naked eye. Therefore, research about the application of digital image colorimetry has bloomed in the past decade. These studies have tried to quantify the metal cations [14,15,16,17], nitrate (trinitrotoluene (TNT) [18], ammonium nitrate [19]), phosphorus [17], chlorine [14,20,21] in water through digital images by using either camera, smartphone or webcams, but without a comparable method to correct light influence or using different color values, which makes it difficult to make a comparison. Details of the literature are shown in Table S1.

In this article, a water color image acquisition system, inspired by colorimetric measurements used in analytical chemistry, was set up to capture RGB image data of artificial water samples. This technique permitted obtaining a water color index from a water sample with known concentrations of OACs, other than smartphone applications or satellite observations, which measure the OACs indirectly [22]. Total organic carbon analysis (TOC) and UV-VIS spectrophotometry were applied to validate the chemical concentration of water components. Our low-cost setup can be used in the quantitative research of water quality and as a comparison of citizen science or satellite observations. If the quantitative determination of OACs can be improved, the aforementioned applications could be easily applied to citizen science [1].

## 2. Materials and Methods

Two kinds of domain OACs—phytoplankton and CDOM—in natural water were investigated in our study. They are substituted by using algae species *Neochloris oleoabundans* obtained from AlgaePARC, and commercial humic acid (Sigma-Aldrich (St. Louis, MO, USA), technical), respectively. The details of how to prepare our aqueous samples are elaborated in the Supplementary Materials.

We designed a low-cost digital setup to measure the water color with known accuracy. Images of the aqueous samples were taken by a digital camera (SONY ILCE-5000L). Through a protocol of light and device calibration, the color indices (xy chromaticity, hue value) were extracted from the pictures. Humic acid as a proxy for CDOM was used to test and adjust our setup. A total organic carbon analyzer (TOC-L, Shimadzu) was used to validate humic acid concentration. Further applications were repeated for the algae pigments and validated by UV-VIS spectrophotometer (VWR). The relationship between color indices and algae pigment/humic acid concentrations was then investigated.

### 2.1. Digital Colorimetry Setup

**Figure 1:**
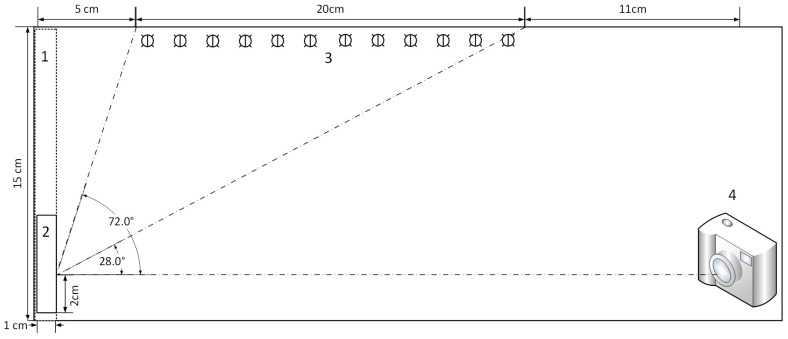
Illustration of colorimetry setup.

There are five essential parts in our acquisition system. The type and size of each part is listed below and illustrated in Figure 1. The budget of the setup was listed in Table S2.

1. A sample holder in black background (No.1 in the illustration. It is made by a black foam and kept at a fixed position.);
2. A cuvette for samples (No.2 in the illustration.);
3. A light source (No.3 in the illustration. 12 LED bulbs (6000 K, 220 lumens, YPHIX${}^{®}$) at the middle-top of the box, which have an adjustable light illumination with a remote controller. The emitted light spectra are shown in Figure S1.);
4. A camera (No.4 in the illustration. The camera used here was a SONY ILCE-5000L, with an APS-C CMOS sensor. It was placed at the right side of the bottom.);
5. A digital lux meter (PeakTech P5025) was used to measure the illumination in the box. The position of the digital lux meter was at the same position as the sample, and the illumination was measured before taking photos.

### 2.2. Light Condition and Camera Setting

A 6000k LED light was used as the light source because it is closer to natural solar illumination. In order to be used in different circumstances, different light conditions and camera settings were tested in our experiment. After some preliminary tests, the light was finally set for two conditions: 510 and 1010 Lux. The aperture and shutter speed of the camera were fixed. The main adjustment of the camera was the ISO rating. The ISO was tested at 400 and 800. A device-independent color space CIE-XYZ [23] was used for the correction of camera differences.

### 2.3. Image Correction Protocol

**Figure 2:**
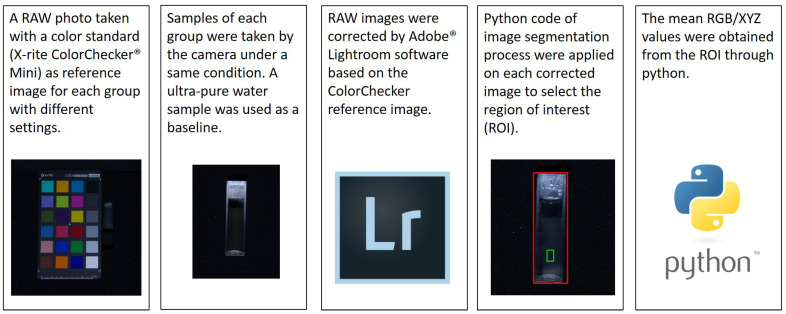
Image correction process by using a color standard (X-rite ColorChecker${}^{®}$ Mini) and color space transform.

In order to have a repeatable and comparable measuring protocol, the image color indices were obtained through a standard procedure, including pre-processing and correction processes, as shown in Figure 2.

At first, the water color images were taken while a color standard X-rite ColorChecker${}^{®}$ Mini was taken under the same circumstance (light illumination and camera setting). We chose to collect RAW image format instead of JPEG format since JPEG compression may lose information during data processing and compression and is difficult to calibrate. Moreover, RAW data shows high linearity and is not affected by white balance [24]. Therefore all the images were saved in RAW data format and then corrected by Adobe${}^{®}$ Lightroom software with the ColorChecker standard.

Then, a self-developed python code was used to extract the RGB values and CIE XYZ values from the sample region of interest (ROI). The details of selecting ROI and the algorithm of converting RGB values into xy chromatic diagram and hue angle were explained in the subsections.

#### 2.3.1. Region of Interest

After correcting the differences caused by different light illuminations and camera settings, the color information was extracted from the backgrounds by an image segmentation process. A python code that included threshold and region-based segmentation was applied to the images. After selecting the interest region, thousands of pixel data were collected. A confidence interval was used in the python code to refine the mean of pixel results within the region of interest.

#### 2.3.2. True Water Color Chromaticity Retrieval

The definition of true water color here is the spectral responses of three cones in the human vision system (or linear transform of the cone responses). Therefore, the international standard CIE XYZ color space, also known as spectral tristimulus values (X/Y/Z), has been introduced to quantify image color. Color matching functions are used to tell the sensitivity of the human eye to three primary colors (Red, Green, Blue) and serve as weighting functions [25]. The algorithms to convert spectrum information into human color perception are illustrated as follows:

(1) $$X=\int I\left(\lambda \right)\times \bar{x}\left(\lambda \right)d\lambda ;$$

(2) $$Y=\int I\left(\lambda \right)\times \bar{y}\left(\lambda \right)d\lambda ;$$

(3) $$Z=\int I\left(\lambda \right)\times \bar{z}\left(\lambda \right)d\lambda .$$

where $\bar{x}\left(\lambda \right),\bar{y}\left(\lambda \right),\bar{z}\left(\lambda \right)$ are the color matching functions from CIE (Comité International d’Eclairage) tables. I(λ) is the intensity of the visible band. Then the x,y,z coordinates are calculated through:

(4) x=X/(X+Y+Z);

(5) y=Y/(X+Y+Z);

(6) z=Z/(X+Y+Z).

The hue angle is defined the same as in van der Woerd [25]:

(7) $$\alpha =\arctan(y-y_{w},x-x_{w})modulus2\pi$$

where $x_{w}$ and $y_{w}$ are the coordinates of the white point $\left(x_{w}=y_{w}=z_{w}=1/3\right)$.

In previous research [25,26], X/Y/Z values are the product of the illumination multiplied by the remote sensing reflectance, which comes from limited spectral bands of satellite products (such as SeaWiFS, MODIS, Sentinel-3 OLCI). These bands are limited in number and are with a wide band width, which may cause large errors while reconstructing of hue angle [7]. However, our hue angle was calculated from the spectral response of each wavelength over the visible spectrum. Therefore, it avoids the inaccuracy caused by the band limitation of the satellite sensors.

## 3. Results

### 3.1. Image Correction

**Figure 3:**
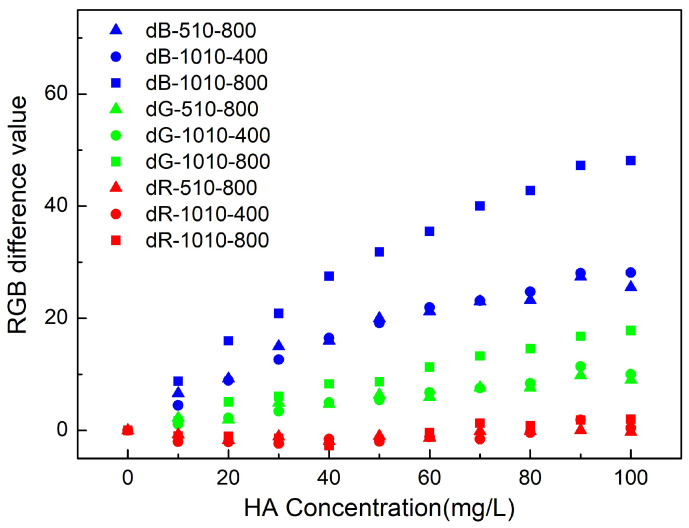
The RGB difference value calculated from the image at different light illuminations (i.e., 510 and 1010 Lux) and different camera ISO (i.e., 400 and 800) of dissolved humic acid at gradually increasing concentrations (ultra-pure water sample as the blank). R denotes red channel, G denotes green channel, B denotes blue channel. After the color channel, the light illumination comes after, and the last is the camera ISO setting. For instance, dR-510-800 denotes the difference of red channel derived from the image taken at 510 Lux illumination with camera setting at ISO800. (The legends in subsequent figures follow the same pattern.)

The results of RGB differences under two different light illuminations (510 and 1010 Lux) and two camera ISO settings (400 and 800) are shown in Figure 3. The RGB value of ultra-pure water is the base value, and the difference is calculated by the base RGB value minus the sample RGB value. As can be seen from the figure, changing the concentration of humic acid has the most effect on the blue band, then the green band and the least on the red band. This trend corresponds to the absorption spectra of the samples at visible bands (see Figure S3 in Supplementary Materials).

When the light condition or camera setup changes, RGB values are affected even after the correction of the light illumination by the ColorChecker Classic tool. The Blue band is the most influenced by the light condition, followed by the green band, and then the red band. Since the future application of this technique might be adopted under different illumination conditions with different cameras, the device-dependent RGB values could be hardly used for quantitative OACs in water.

**Figure 4:**
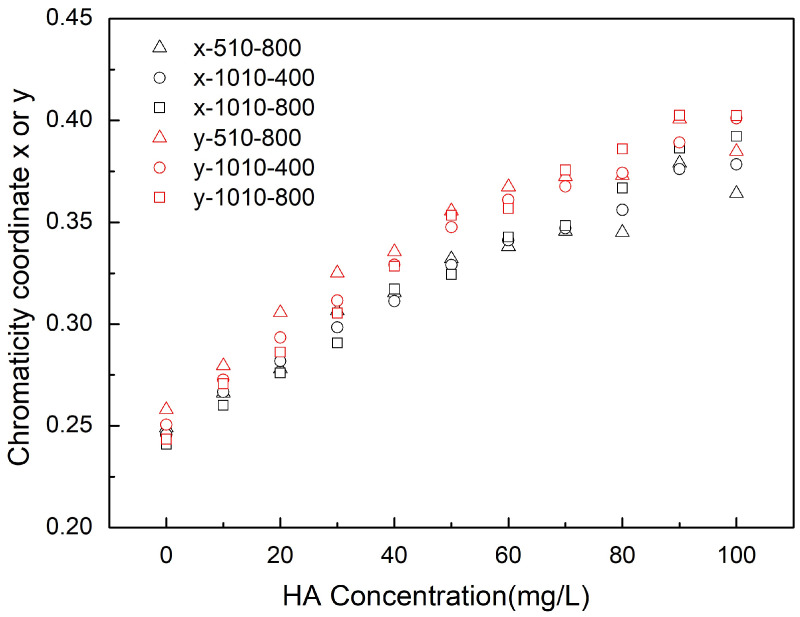
The xy chromatic diagram derived from RGB at different light illuminations (i.e., 510 and 1010 Lux) and different camera ISO (i.e., 400 and 800) of the dissolved humic acid at gradually increasing concentrations.

To further eliminate the influence from light and camera properties, the color space was converted from RGB to xy chromaticity coordinates. The difference of xy values of different concentrations with xy values of water blank is shown in Figure 4. It displays that the xy values also have a near-linear relationship with the concentration but are less impacted by the light conditions or camera settings. The mainstream approach to spectra illustration of CDOM is based on a traditional exponential decay equation proposed by Bricaud et al. [27]. The slope calculated from the absorption coefficient of humic acid (Figure S3) ranges from 0.010–0.011.

From Figure 4, it can be seen that the xy coordinate values under different light conditions and camera settings are similar at low concentrations, and they show a linear relationship with the concentration. Then, the line deviates at a higher concentration. For instance, at 1010 Lux and ISO 800, the linear relationship flattens out at about 80 mgC/L humic acid. The reason for this may be that the absorption of higher concentrations was saturated. Furthermore, this phenomenon could also be observed under a lower illumination (510 Lux) at a lower concentration (about 60 mgC/L). It is shown in the figure that the xy values at 510 Lux (triangle points) are lower than the values at 1010 Lux (square points). Therefore, a suitable light illumination should be chosen carefully. A low illumination will cause an earlier saturation, while a high illumination will cause overexposure of the camera. This is a typical issue in real-world water color observation by small cameras.

### 3.2. xy Color Space Correlation with the Concentration

**Figure 5:**
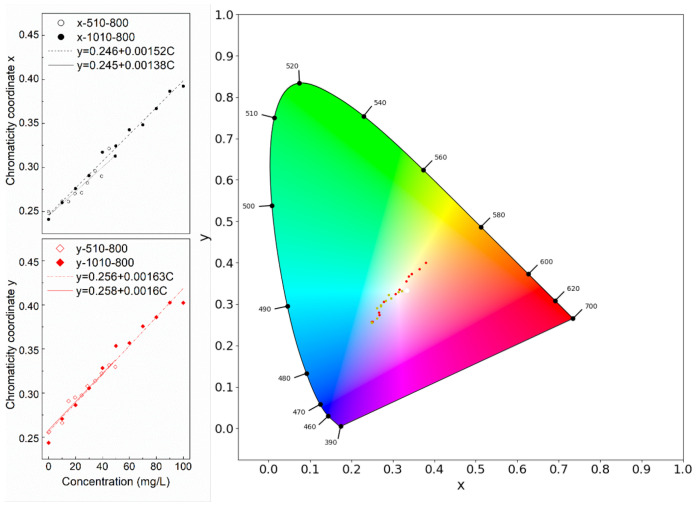
The xy values of different humic acid concentrations in the xy chromatic diagram. The left figure shows a linear relationship simulation between chromaticity x or y values and humic acid concentration.

Furthermore, two groups of the xy values of humic acid in a chromatic diagram were plotted in Figure 5, and their linear fitting were disclosed at the left. As shown in the figure, the linear fit of xy derived from different ISO and illumination almost overlap. The slight deviation of x might be caused by a systematic validation error from the TOC analysis. With the concentration increasing, the dots in the chromatic diagram moved from the blue region toward the white point and then to the orange region. The point about 50 mgC/L is the closest to the white point.

**Figure 6:**
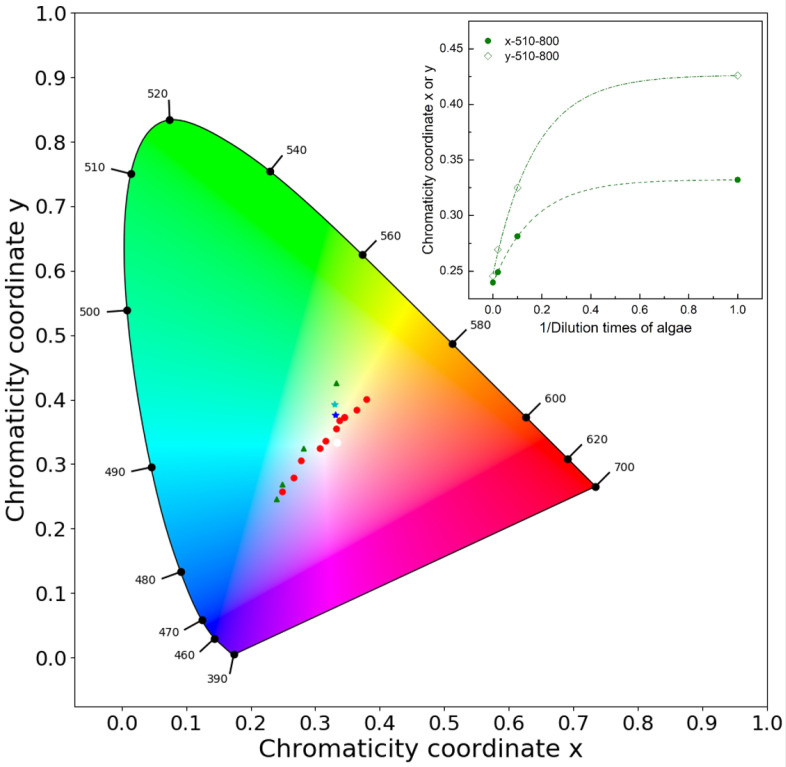
The xy values of extracted two mixed solutions(cyan and blue star dots), algae pigment (green triangle dots) and humic acid (red round dots) in the xy chromatic diagram. The manipulated variables are 510 Lux and ISO 800. The inlaid figure is an exponential relationship simulation between xy values and the inverse values of the dilution times of algae pigment.

The same process was applied to algae extraction under 510 Lux illumination and ISO 800. The original extraction was diluted 10 to 50 times. Three different concentration solutions and one 10% acetone control sample were pictured. Their xy values with inverse values of the dilutions are plotted in the xy chromatic diagram (Figure 6). The inlaid figure shows that the absorption of algae is saturated after 10 times dilution and an exponential relationship with a very high concentration of algae pigment is observed.

Finally, two samples with mixed algae pigment and humic acid were made and tested in the colorimetry setup. They were plotted in Figure 6. The xy values of pure algae pigments and humic acid were plotted in the same figure for comparison. Their absorption spectrum is available in the Supplementary Materials (Figures S2–S4). The cyan star dot is with 0.25 times algae solution plus 37.5 mgC/L humic acid. The blue star dot is with 0.1 times algae solution plus 45 mgC/L humic acid. As can be seen, these two points were in between the plots of algae extraction and the plots of humic acid. If they were considered as a single component, not as mixed components, the estimated concentration of the single component might be overestimated. It means that if the blue dot were identified as original from humic acid only, the derived concentration would be around 60–70 mgC/L, not 45 mgC/L as it was.

### 3.3. hue Angle Correlation with the Concentration

**Figure 7:**
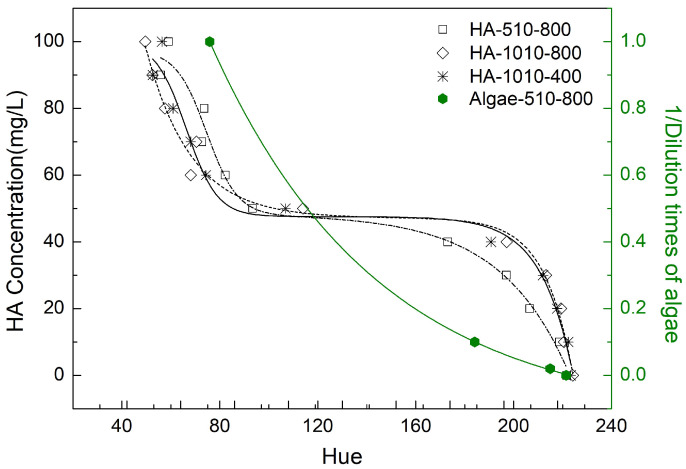
The hue angle of pure humic acid (in black) and algae extraction (in green) at different concentrations.

We calculated the hue angle based on Equation (7). As seen in Figure 7, the hue angle has a negative correlation with OACs’ concentration. Moreover, the hue angle of humic acid could be fitted with S-shape curves which is similar to previous studies [7,28]. The turning point happens around a concentration of 50 mgC/L. This could relate to the transmission of xy to hue. The supporting evidence is that 50 mgC/L is the closest to the white point (see Figure 5). Both of the substitutions for phytoplankton and CDOM show a negative correlation between concentration and the hue angle. This corresponds to the results of the hue angle derivations from satellite sensors [7,8,28].

## 4. Discussion

This paper showed a design of a low-cost digital camera colorimetry setup and a method to correct the bias caused by different light illumination conditions and camera settings. We built this simple experimental setup in order to mimic smartphone setups, typically used today by many people (such as citizen observatories [1,2,3]) to contribute to environmental and global water quality monitoring [8]. With our simple design, we obtained water color images, which could be used to calculate water color indices. By using a standard measuring and data-processing protocol for calibrating the images, we could relate the water color observations and derived color indices (such as xy chromaticity, hue angle) to the chemical concentration of OACs in water, quantitatively.

As we show in Figure 3, the RGB color space used as a quantitative parameter can be unreliable due to the uncertainties introduced by using a different camera or lens [24]. However, the effect of the different light illuminations and camera settings can be corrected by the image correction procedure we introduced in this paper. By using the xy chromatic diagram, the camera differences can be accounted for and removed (see Figure 4).

After the correction, we could use the xy coordinates as a color index to quantify the concentration of the OACs. We explored the relationship between the different color indices and different concentrations of two common OACs, i.e., phytoplankton pigments and CDOM. Concentration patterns can be observed in the xy chromaticity and water color diagrams (see Figure 5 and Figure 6), which brings digital color observations, such as smartphone water color pictures, closer to determining water quality constituents. However, we do not yet have an immediate solution to quantify one component from a mixed solution, and more experimental research on this is needed. However, it provides robust information that is simply using digital colorimetry to determine OACs concentration from complex waters might lead to an overestimation if only a single component is considered.

As for the hue angle, we found that the concentration of humic acid and algae pigments fits a descending relationship with the hue angle. In previous studies [7,28], which have the same definition of the hue angle as ours, researchers found a negative relationship between the hue angle and absorption coefficient based on satellite data. According to the Beer–Lambert law, absorption is positively associated with concentration. Therefore, our results, in relation to component concentrations, confirm previous studies.

We only considered light absorption here to keep an idealized simulation. As in our sample, we did not introduce solid or colloidal particles, which would cause light scattering. To further develop the low-cost digital colorimetry for real water body monitoring, more efforts should be put on studying and combining the effects of particles on light attenuation for the derivation of water color from water color pictures, and then scaling up to a large experimental size.

However, we think that evaluating the concentration of a mixed solution still remains challenging by using digital RGB images only. We also mention that there remains a possibility that the same visible water color is generated by different chemical compounds and/or concentrations. As can be seen in most cases, it is still hard to do the inverse simulation of water color. For instance, mixed solutions are typically found in Case II water (such as coastal areas) and water bodies affected by land-based runoff processes, which lead to combined CDOM and SPM attributions. Nevertheless, water colour observations have a record in history and are useful for classifying different water ecosystems through remote sensing. Therefore, it is crucial to investigate further what mixture of OACs can yield the same or similar color.

## 5. Conclusions

Our work is a study combining a low-cost image acquisition setup with advanced quantitative laboratory measurements. A correcting process through RAW image and the ColorChecker Classic tool can be recommended since the ColorChecker Classic tool is cheap and commonly used by photographers. On the one hand, similar to scaling up the experiment size or measuring in situ, acquiring uniform outputs after implementing light conditions and digital camera correction methods is essential for comparing different research results in future work. On the other hand, easy access and low cost of these tools or setups promote citizen science observation.

## Data Availability

The data presented in this study are available on request from the corresponding author.

## Supplementary Materials

The following are available at https://www.mdpi.com/article/10.3390/s21206699/s1. A low-cost digital colorimetry set-up to investigate the rela-tionship between water color and its chemical composition.
