# Supplementary material for: A Low-Cost Digital Colorimetry Setup to Investigate the Relationship between Water Color and Its Chemical Composition
Source: Sensors (Basel). 2021 Oct 9;21(20):6699. doi: 10.3390/s21206699 (PMC8541544; doi:10.3390/s21206699)
Supplement: Supplementary file 1 [file sensors-21-06699-s001.zip › sensors-1357949-supplementary.pdf]

sensors

MDPI

Supplementary Materials

A low-cost digital colorimetry set-up to investigate the relationship between water color and its chemical composition: supplemental document

Ruosha Zeng [1,*], Chris M. Mannaerts [1] and Zhehai Shang [2]

[1] Department of Water Resources, Faculty of Geo-information Science and Earth Observation, University of Twente, 7514AE Enschede, the Netherlands
[2] Institut de la Mer de Villefranche, 181 Chemin du Lazaret, 06230 Villefranche-sur-Mer, France
* Correspondence: r.zeng@utwente.nl

1. LABORATORY EQUIPMENT

Absorption spectra were measured with a VWR UV-6300PC double beam UV-VIS spectrophotometer using a 1 cm quartz cuvette. Humic acid concentration measurements were performed using a Shimadzu TOC-L CPH analyzer. 90 μL injection of water samples were combusted at 680°C. Then, the difference method by subtracting IC(inorganic carbon) from TC(total carbon) was used for calculating dissolved organic carbon (DOC).

2. CHEMICALS AND SAMPLE PREPARATION

All of the water samples and solutions were prepared with ultra-pure water(Milli Q). The TOC standard stock solutions(1000mg/L) were prepared by dissolving 0.8833g sodium carbonate (for Inorganic carbon, Nacalai Tesque, INC.) and 0.2127g potassium hydrogen phthalate (for Organic carbon, Nacalai Tesque, INC.) with 100 mL ultra-pure water. Pigments were extracted using 90% acetone (technical pure, Boom BV). The algae were mixed with water to form a 5 wt.% slurry which was stored at -22 °C in a freezer.

A. Algal pigment extraction

The extraction procedure was based on a protocol method 446.0 [1]. First, 2 mL 5% wt algae slurry was filtered through a Whatman GF/C glass-fibre filter. Second, repeatedly freezing and thawing the filter were performed to break down the cells of algae. During this process, the algae were kept in a dark environment. Third, 1mL 1%wt MgCO3 water suspension and 4 mL acetone were added into the grinder and then ground to extract the pigment. The final slurry was poured into a 15 mL centrifuge tube. An add-on 5 mL acetone was used to rinse the pestle and the grinder and moved into the same tube. Added a little acetone to make a 10mL solution in total. The tube was placed in an ultrasonic bath for 5min with water and ice in a beaker and stored at 4°C for 2-24 h. The last step was centrifuging the tube at 3500 r/min for 15 min to separate and then carefully pulled out the clear liquid part. In the end, chlorophyll-a and other pigments of algae were extracted by 90% acetone.

B. Dissolving humic acid

Humic acid is unsolvable in water, but it dissolves in an alkaline solution. Here we used sodium hydroxide at pH 9. We kept it stirring overnight and then filtered it. The pH was measured after filtering, then modified back to 7 with hydrochloric acid. The final solution was stored at 4 °Cin a fridge. For gradual concentration reduction, ultra-pure water was used for diluting. The concentration was measured by a TOC analyzer.

3. TABLES

Table S1 is a literature summary of digital colorimetry used in analytical chemistry.

Table S2 shows a list of budget for a low-cost digital colorimetry set-up.

Table S1. Digital image based analysis of chemical compounds in water.

Analyte	Color space	Range	RSD(%)	Ref.
Ca(II)	RGB	0.2-2.0 mg/L	5.5%	[2]
Fe(III); Chlorine	L*a*b	<2.0 mg/L; <1.0 mg/L	3.4%-10%	[3]
Chlorine	RGB	0.3-1.0mg/L	7%	[4]
Al(III);Cr(VI)	RGB	10-600 $\mu g/L$; $10-300\mu g/L$	1.5%;1.7%	[5]
Ammonium nitrate	RGB	5-250mg/L	0.03%-2.54%	[6]

Table S2. Estimated price for a low-cost digital colorimetry set-up.

Number	Equipment	Cost
1	Box	€50
2	Disposable Cuvettes	€30 for 100
3	LED light strip	€50
4	Cameras or smartphone	No extra cost*
5	A color standard	€65

*The camera was from the laboratory. Any camera in hands could be used for this set-up.

4. FIGURES

Figure S1 shows the spectrum of the light source using in our set-up.

Figure S2, S3, and S4 are the absorption spectra of mix solution, humic acid samples, and algae extraction respectively.

The absorption coefficient was derived from the absorbance measured by the VWR spectrophotometer. The transform equation of absorption coefficient $a(\lambda)$ is:

$$a(\lambda) = 2.303 \times A(\lambda)/l \tag{1}$$

$A(\lambda)$ is the absorbance; l is the cuvette length which is 1cm.

The traditional exponential decay equation by Bricaud et al. [7] of CDOM slope (S) is:

$$a(\lambda) = a(\lambda_0)\exp[-S(\lambda - \lambda_0)] \tag{2}$$

$a(\lambda), a(\lambda_0)$ are the absorption coefficient at wavelength λ, λ_0, respectively. Here, λ_0 is 350nm.

The absorption of pure algae extraction ("C" in fig. S4) has exceeded the detecting limits of our spectrophotometer.

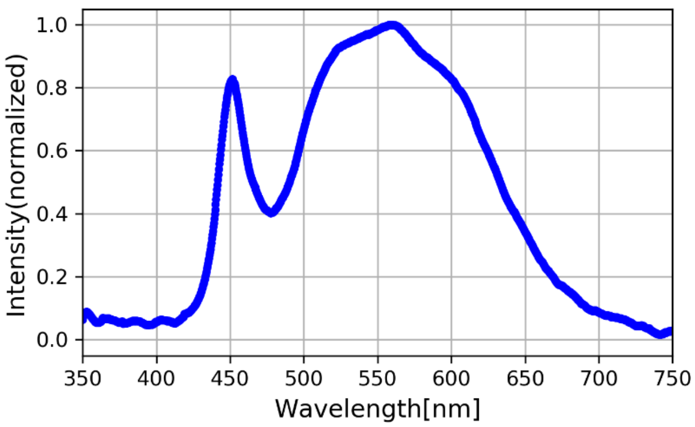

Figure S1. Normalized light intensity spectrum of the source commercial 6000k LED light.

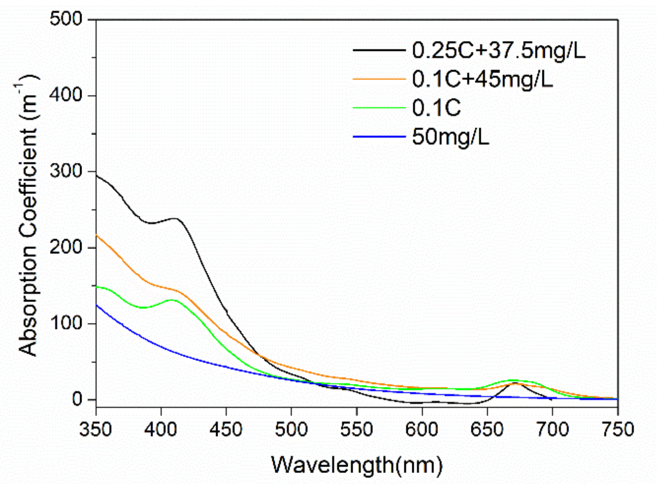

Figure S2. Absorption spectra of the mix samples. A humic acid sample (50mg/L) and a pure algae sample (10 times diluted) are plotted here for comparison.

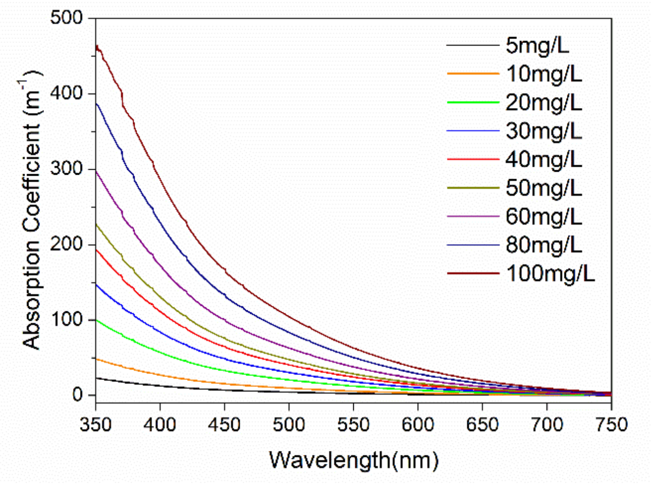

Figure S3. Absorption spectra of humic acid samples.

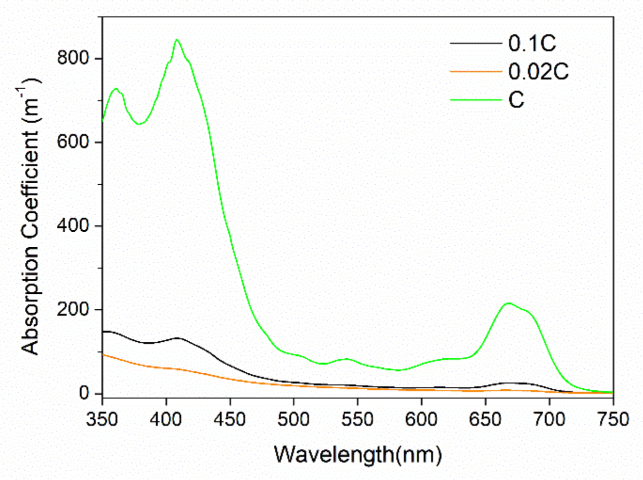

Figure S4. Absorption spectra of the algae samples.

REFERENCES

1. Arar, E. J., Method 446.0: In Vitro Determination of Chlorophylls a, b, c + c and Pheopigments in Marine And Freshwater Algae by Visible Spectrophotometry. U.S. Environ. Prot. Agency, 1997, Washington, DC EPA/600/R-15/005.
2. Lopez-Molinero, A.; Tejedor Cubero, V.; Domingo Irigoyen, R.; Sipiera Piazuelo, D., Feasibility of digital image colorimetry--application for water calcium hardness determination. *Talanta* **2013**, *103*, 236–244.
3. Suzuki, Y.; Endo, M.; Jin, J.; Iwase, K.; Iwatsuki, M., Tristimulus Colorimetry Using a Digital Still Camera and Its Application to Determination of Iron and Residual Chlorine in Water Samples. *Anal. Sci.* **2006**, *22*, 411–414.
4. Sumriddetchkajorn, S.; Chaitavon, K.; Intaravanne, Y., Mobile device-based self-referencing colorimeter for monitoring chlorine concentration in water. *Sens. Actuators, B* **2013**, *182*, 592–597.
5. Andrade, S. I. E.; Lima, M. B.; Barreto, I. S.; Lyra, W. S.; Almeida, L. F.; Araújo, M. C. U.; Silva, E. C., A digital image-based flow-batch analyzer for determining Al(III) and Cr(VI) in water. *Microchem. J.* **2013**, *109*, 106–111.
6. Choodum, A.; Boonsamran, P.; NicDaeid, N.; Wongniramaikul, W., On-site semi-quantitative analysis for ammonium nitrate detection using digital image colourimetry. *Sci. Justice* **2015**, *55* , 437–445.
7. Bricaud, A.; Morel, A.; Prieur, L., Absorption by dissolved organic matter of the sea (yellow substance) in the UV and visible domains. *Limnol. Oceanogr.* **1981**, *26*, 43–53.
